# Supplementary material for: Combination of Cisplatin and Irradiation Induces Immunogenic Cell Death and Potentiates Postirradiation Anti–PD-1 Treatment Efficacy in Urothelial Carcinoma
Source: Int J Mol Sci. 2021 Jan 7;22(2):535. doi: 10.3390/ijms22020535 (PMC7825793; doi:10.3390/ijms22020535)
Supplement: Supplementary file 1 [file ijms-22-00535-s001.zip › Supplementary materials/Figure S1.pptx]

## Slide 1
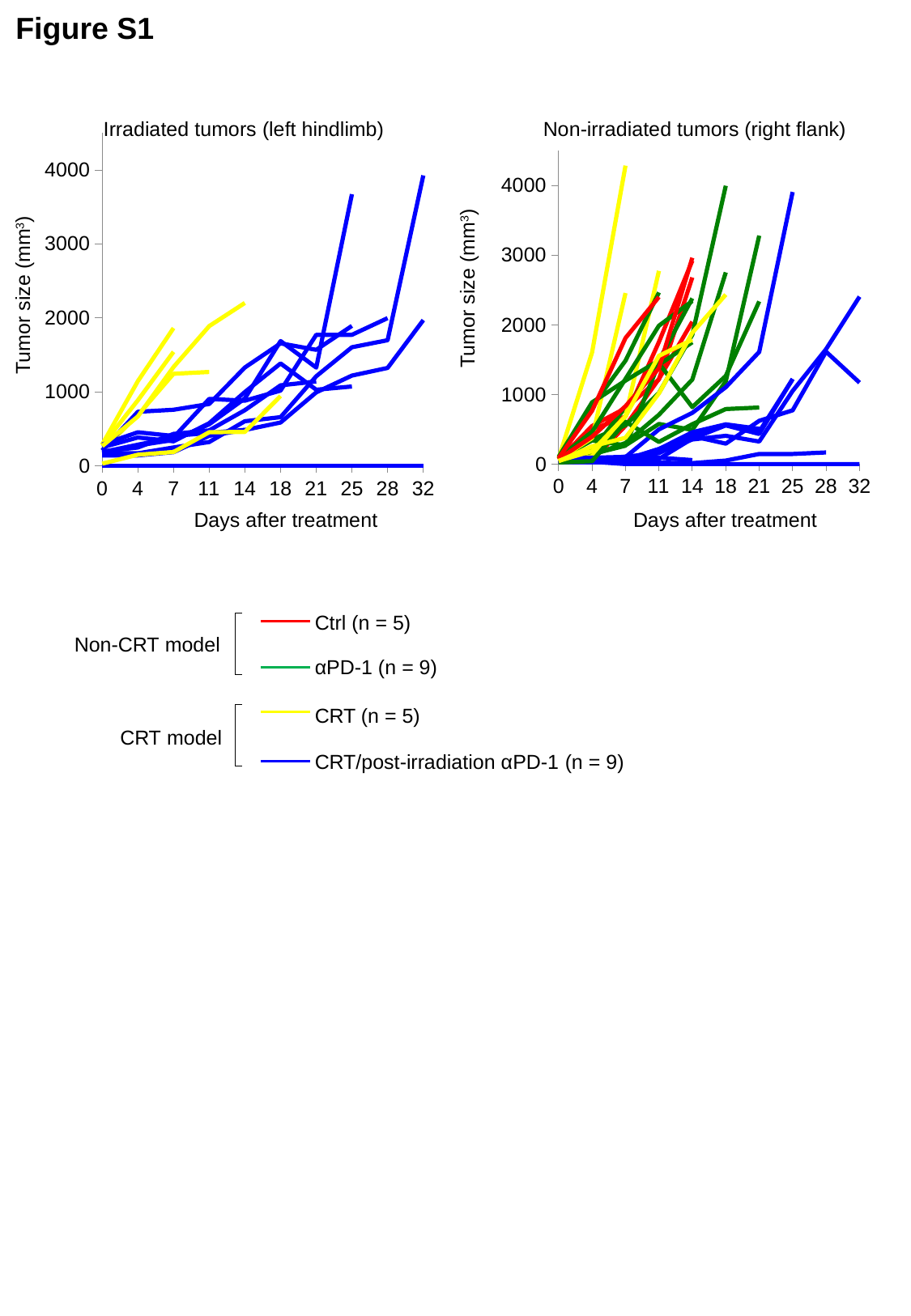

Figure S1
Irradiated tumors (left hindlimb)
[unsupported chart]
Tumor size (mm3)
Days after treatment
Non-irradiated tumors (right flank)
[unsupported chart]
Tumor size (mm3)
Days after treatment
Ctrl (n = 5)
Non-CRT model
αPD-1 (n = 9)
CRT (n = 5)
CRT model
CRT/post-irradiation αPD-1 (n = 9)
