# Supplementary material for: Combination of Cisplatin and Irradiation Induces Immunogenic Cell Death and Potentiates Postirradiation Anti–PD-1 Treatment Efficacy in Urothelial Carcinoma
Source: Int J Mol Sci. 2021 Jan 7;22(2):535. doi: 10.3390/ijms22020535 (PMC7825793; doi:10.3390/ijms22020535)
Supplement: Supplementary file 1 [file ijms-22-00535-s001.zip › Supplementary materials/Figure S2.pptx]

## Slide 1
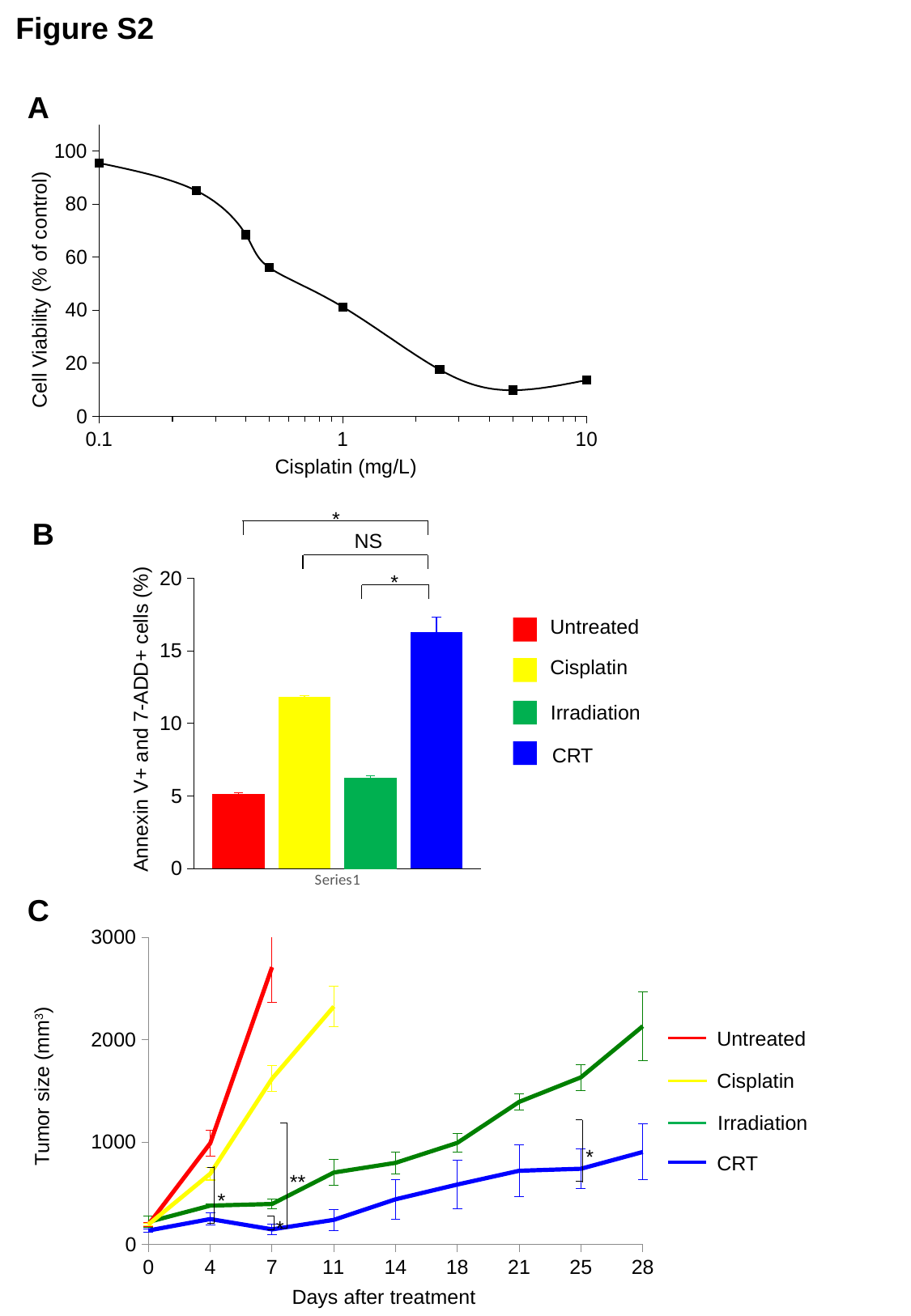

Figure S2
A
### Chart
| Category | |
|---|---|Cell Viability (% of control)
Cisplatin (mg/L)
*
B
NS
### Chart
| Category | | | | |
|---|---|---|---|---|
| | 5.1433333333333335 | 11.833333333333334 | 6.22 | 16.266666666666666 |*
Untreated
Cisplatin
Irradiation
Annexin V+ and 7-ADD+ cells (%)
CRT
C
[unsupported chart]
Untreated
Tumor size (mm3)
Cisplatin
Irradiation
*
CRT
**
*
*
Days after treatment
