# Supplementary material for: Combination of Cisplatin and Irradiation Induces Immunogenic Cell Death and Potentiates Postirradiation Anti–PD-1 Treatment Efficacy in Urothelial Carcinoma
Source: Int J Mol Sci. 2021 Jan 7;22(2):535. doi: 10.3390/ijms22020535 (PMC7825793; doi:10.3390/ijms22020535)
Supplement: Supplementary file 1 [file ijms-22-00535-s001.zip › Supplementary materials/Figure legends of supplementary figures.doc]

**Figure S1** MB49 tumor growth curves of irradiated left hindlimb (*left*) and non-irradiated right flank tumors (*right*) in all mice in the CRT and Non-CRT models.

**Figure S2** The combination of cisplatin and irradiation showed strong cytotoxic effects on MB49 cells. A, Cytotoxicity assay for MB49 cells treated with eight different cisplatin concentrations (0, 0.25, 0.4, 0.5, 1, 2.5, 5, 10 mg/L; n = 6 wells/concentration). B, MB49 cells were treated with cisplatin at 0.6 mg/L and/or irradiated with a single fraction of 10 Gy. Cells were collected two days after treatment and the percentage of apoptotic cells were examined by flow cytometry (n = 3/group). C, Six- to seven-week-old mice were subcutaneously inoculated with MB49 cells (2 × 10^6^, in matrigel) in the left hindlimb. Seven days after inoculation, mice were treated with cisplatin at 3 mg/kg and/or irradiation with a single fraction of 10 Gy to the left hindlimb. MB49 tumor growth curves of tumors in the left hindlimb. All data are shown as the mean ± standard error of the mean (SEM). Asterisks indicate *p-*values comparing two groups, as indicated in the figure. NS, not significant; *, *p* < 0.05; **, *p* < 0.01.
